# The Role of Traditional and Reverse Algorithms in the Diagnosis of Syphilis in HIV-Infected Individuals: A Case Study on Istanbul

**DOI:** 10.3390/diagnostics15030365

**Published:** 2025-02-04

**Authors:** Gizem Yapar, Muammer Osman Köksal, Kutay Sarsar, Pınar Soğuksu, Mehmet Demirci, Eray Yurtseven, Murat Hakan Kır, Aytaj Allahverdiyeva, Arif Atahan Çağatay, Ali Ağaçfidan, Hayriye Kırkoyun Uysal

**Affiliations:** 1 Department of Medical Microbiology, Istanbul Faculty of Medicine, Istanbul University, 34093 Istanbul, Turkey; gizem.yapar@ogr.iu.edu.tr (G.Y.); muammerosmankoksal@istanbul.edu.tr (M.O.K.); kutay.sarsar@istanbul.edu.tr (K.S.); soguksup@yahoo.com (P.S.); dr.aytach92@gmail.com (A.A.); aagacfidan@hotmail.com (A.A.); 2 Department of Medical Microbiology, Institute of Health Sciences, Istanbul University, 34126 Istanbul, Turkey; 3 Department of Medical Microbiology, Faculty of Medicine, Kırklareli University, 39100 Kırklareli, Turkey; mdemirci1979@gmail.com; 4 Department of Biostatistics, Istanbul Faculty of Medicine, Istanbul University, 34093 Istanbul, Turkey; eyurt@istanbul.edu.tr; 5 Department of Infectious Diseases and Clinical Microbiology, Istanbul Faculty of Medicine, Istanbul University, 34093 Istanbul, Turkey; murathakankir@gmail.com (M.H.K.); acagatay@istanbul.edu.tr (A.A.Ç.)

**Keywords:** HIV, syphilis, syphilis diagnostic algorithms, sexually transmitted diseases

## Abstract

**Background/Objectives:** *T. pallidum* subsp. *pallidum* is a spirochete that only causes disease in humans as the causative agent of syphilis. HIV and syphilis have common transmission routes. In the present study, our aim was to evaluate the presence of syphilis coinfection in people living with HIV using conventional and reverse algorithms and to investigate its effects on laboratory parameters. **Methods**: The traditional algorithm for syphilis begins testing with the non-treponemal test. If the non-treponemal test is reactive, a treponemal test is then used to confirm syphilis infection. The reverse algorithm for syphilis begins testing with the treponemal test. If this test is reactive, then a non-treponemal test is performed. When the non-treponemal test is non-reactive, a second treponemal test is performed. **Results**: A total of 200 patients were included in the study. While 22 patients were determined to be syphilis-positive using the traditional algorithm, 37 patients were determined to be syphilis-positive using the reverse algorithm. Most of the patients who had syphilis coinfection were homosexual. **Conclusions:** It was found that syphilis coinfection had no effects on CD4+ T-lymphocyte and CD8+ T-lymphocyte values, CD4/CD8 rates, and HIV RNA amounts. People living with HIV must be screened and followed up for syphilis and other sexually transmitted diseases at certain intervals.

## 1. Introduction

Syphilis is a sexually transmitted infection (STI) or congenitally acquired disease caused by *Treponema pallidum subs. pallidum*. Although it has a definite cure when treated early, in untreated cases, it might progress and cause serious life-threatening complications and internal organ involvement. It might also present with various clinical symptoms such as genital ulceration, rash, neurological dysfunction, and stillbirth. There is no natural immunity against the infection [1,2,3].

The disease has almost always had a high incidence in developing countries, and the number is particularly striking in low- and middle-income countries where it is endemic. The problem is more pronounced among young people and young adults in low socioeconomic groups who are sexually active in developed countries. The disruptions in prevention, testing, and treatment services for sexually transmitted infections (STIs) during the COVID-19 pandemic have led to a resurgence of STIs [4,5].

According to the CDC, 203.500 cases of acquired syphilis and 3.755 cases of congenital syphilis were reported in 2022. These figures show a 17% increase in acquired syphilis and a 30.6% increase in congenital syphilis compared to 2021, which means that syphilis poses a public healthcare concern despite COVID-19 and the under-reporting of cases [6].

Despite the availability of simple diagnostic tests and the effectiveness of penicillin treatment, syphilis remains a global public healthcare concern, particularly among men who have sex with men (MSM) in high- and middle-income countries [7].

Epidemiological studies report that the incidence of syphilis among men in the United States has more than tripled since 2000. The estimated prevalence and incidence of syphilis vary greatly among regions or countries, and it is most common in Africa, with the highest prevalence and >60% of maternal syphilis cases [7,8,9]. Based on data from the European Center for Disease Prevention and Control (ECDC), syphilis rates are 9-fold higher in men than in women, peaking in men in the age group of 25–34 years old [10]. Based on data from the General Directorate of Public Health of the Ministry of Health, 3533 cases have been reported in Turkey. The majority of these are male and are between the ages of 25 and 29. In women, there are small differences, with the highest number of cases being between the ages of 20 and 24 (120 cases). It is striking that the majority of cases are detected in young people [11].

Syphilis is most commonly transmitted through sexual contact. However, transmission from mother to child is also seen. Most cases of transmission during pregnancy are considered to be transplacental in the uterus, but, although rare, transmission during birth is also possible [12].

Serological tests are frequently employed in the diagnosis of syphilis in the present day. These tests are divided into two groups: treponemal tests (Treponema pallidum hemagglutination assay (TPHA) and chemiluminescent microparticle enzyme immunological assay (CMIA)) and non-treponemal tests (Venereal Disease Research Laboratory (VDRL) and rapid plasma reagin (RPR)). There is currently no gold-standard diagnostic approach, as both methods have their own advantages and limitations. Two or more methods must be used to avoid misdiagnosis and missed diagnoses during serological screening. Also, a comprehensive judgment must be made using both clinical symptoms and epidemiological history. Moreover, HIV infection has been associated with false-positive and -negative syphilis serology, making its diagnosis and treatment difficult [13,14,15].

Test results are evaluated with one of two algorithms (the traditional and reverse algorithm). In the traditional algorithm, firstly, RPR (for screening) or a non-treponemal test such as VDRL is used. Positive results are confirmed with a treponemal test. In the reverse algorithm, the treponemal test is used first, followed by a non-treponemal test. Samples with discrepant results undergo a second treponemal test. Both algorithms have their advantages and disadvantages. No one serological test alone can confirm a diagnosis; therefore, all results must be supported by clinical data [16].

Human immunodeficiency virus (HIV) and syphilis have a common transmission route; therefore, they affect similar groups, and the risk of coinfection is high. Coinfection with HIV and syphilis has been increasing globally in recent years. It is thought that there are many reasons for this increase. Improved HIV management, thanks to developed and available anti-retroviral therapy (ART), has led to the re-establishment of sexual networks and increased risk behavior. The use of the Internet to find sexual partners has also contributed to this increase [17]. Ulcers resulting from syphilis infection might increase HIV transmission, and syphilis might increase HIV viral load and reduce CD4+ T-cell count, which might cause faster HIV progression and treatment failure. Moreover, HIV infection has been associated with false-positive and negative syphilis serology, making its diagnosis and treatment difficult. Since detection and treatment are important for transmission, people living with HIV must undergo syphilis screening, and patients diagnosed with syphilis must undergo HIV screening [18,19,20]. It is thought that there are many reasons for this increase. Improved HIV management, thanks to developed and available anti-retroviral therapy (ART), has led to the re-establishment of sexual networks and increased risk behavior. The use of the Internet to find sexual partners has also contributed to this increase [17,18,21].

In the present study, our aim was to emphasize that uncovering the data concerning syphilis coinfection in patients living with HIV is important for public healthcare because of the rapid increase in both infectious diseases in Turkey in recent years. The study also aimed to evaluate the presence of syphilis coinfection in people living with HIV by using traditional and reverse algorithms and to examine its effects on laboratory parameters.

## 2. Materials and Methods

### 2.1. Study Design

This is a prospective study, and it aimed to evaluate the presence of syphilis coinfection and laboratory parameters in patients with HIV infection using the traditional and reverse algorithm. In this study, 200 patients with confirmed HIV infection, over the age of 18, who were followed up in the Department of Infectious Diseases and Clinical Microbiology at Istanbul University, Istanbul Faculty of Medicine, between 2022 and 2024, were included.

### 2.2. Data Collection

Syphilis coinfection was investigated in patients who were referred to Istanbul Faculty of Medicine, Department of Medical Microbiology, HIV Reference Laboratory, between 2022 and 2024 and confirmed to be HIV-positive. The sera of 200 patients over the age of 18 were studied with RPR, TPHA, and CMIA tests, and the results were evaluated by using the traditional algorithm and the reverse algorithm.

In the laboratory, the fourth-generation ARCHITECT HIV Ag/Ab Combo Kit was used for the detection of anti-HIV-1 antibodies on an Abbott Device, following the manufacturer’s instructions. The Western blot method was employed to confirm positive test results (INNO-LIA HIV I/II Score FUJIREBIO). The patients were then followed up, with the monitoring of their HIV RNA Viral Load. The Roche Cobas HIV-1 Assay was conducted by using the Fully Automated 6800 System (Roche Molecular Diagnostics, Pleasanton, CA, USA). This assay targets the gag gene and LTR region (dual target), requires a minimum of 0.655 mL of specimen (0.5 mL plus 0.15 mL of dead volume), and yields quantifiable HIV-1 results ranging from 20 to 10,000,000 copies/mL.

The RPR (Spinreact, Spain), TPHA (Spinreact, Spain), and CMIA (Abbot Diagnostic, Wiesbaden, Germany) tests were performed on the HIV-positive patient sera under investigation with traditional and reverse algorithms. RPR is a non-treponemal agglutination test most commonly employed for the qualitative and semi-quantitative determination of plasma reagents in human sera. TPHA is an indirect hemagglutination test used to detect specific anti-T. pallidum antibodies qualitatively and semi-quantitatively. CMIA is a chemiluminescent microparticle immunoassay used for the qualitative detection of Treponema pallidum antibodies using TpN15, TpN17, and TpN47 antigens.

The immunological parameters of the patients were tested by using the Flow-Cytometry Device, and the results were evaluated. CD4 (helper T-cells) and CD8 (cytotoxic T-cells) were prepared using the lyse wash protocol by using the CD45-FITC/CD4-RD1/CD8-ECD/CD3-PC5 (Beckman Coulter, Brea, CA, USA) monoclonal antibody to determine the absolute and percentage values and CD4/CD8 rates. Flow-count fluorosphere solution was added to the samples to obtain the absolute CD4 and CD8 values. The evaluation of the samples was performed by using the Flow-Cytometry Device (Beckman Coulter Navios Ex) and Navios Ex Software v2.0 according to the manufacturer’s instructions.

### 2.3. Ethics

This study was approved with the permission of Istanbul University’s Medical Faculty Medical Research Ethics Research Organization, Istanbul, on 25 November 2022 with file number 2022\1863. Each participant voluntarily participated in the study and gave informed consent.

### 2.4. Data and Statistical Analysis

The test results were evaluated with one of two algorithms (the traditional and reverse algorithm). In the traditional algorithm, firstly, the RPR (for screening) or a non-treponemal test such as VDRL is used. Positive results are confirmed with a treponemal test. In the reverse algorithm, the treponemal test is used first, followed by a non-treponemal test. Samples with discrepant results undergo a second treponemal test. Both algorithms have their advantages and disadvantages. No one serological test alone can confirm a diagnosis; therefore, all results must be supported by clinical data [16].

The data were analyzed with SPSS 30.0 Software (Chicago, IL, USA) and Microsoft Office Excel. Descriptive statistics were given as frequency (*n*) and percentage (%). Following the statistical evaluation of the data and the summarization of descriptive statistics including the mean, standard deviation, minimum and maximum values, and normality, Student’s *t*-test, and ANOVA were performed.

## 3. Results

### 3.1. The Evaluation of the Demographic Data of the Patients Included in the Study

A total of 200 patients who were referred to Istanbul Faculty of Medicine, Department of Medical Microbiology HIV Reference Laboratory between 2022 and 2024 and confirmed to be HIV-positive were included in this study. Among the 200 patients, 32 (16%) were female and 168 (84%) were male. The average age of the patients was 39.8 (39.1 in women and 39.9 in men). A total of 89 patients were married, 111 were single, 170 lived in Istanbul, and the remaining 30 lived in various cities around Turkey. The education levels of the patients were grouped into primary school (64, 32%), secondary school (8, 4%), high school (58, 29%), and university (70, 35%). The majority of the patients were heterosexual (124, 62%), followed by MSM (60, 30%) and bisexual (15, 7.5%), and 1 patient was transgender. The demographic data of the 200 people living with HIV included in the study are given in Table 1.

### 3.2. Determining Syphilis Coinfection with Traditional and Reverse Algorithms

A total of 200 HIV-positive patient samples were first studied with the RPR test, and positive results were found for 22 patients with the traditional algorithm. The TPHA test was performed on 22 patients, and the results were confirmed.

For the reverse algorithm, 200 HIV-positive patient samples were first studied with CMIA, with positive results found for 38 patients and negative results for 162 patients. The RPR test was used in the 38 patients who tested positive; positive results were found for 22 patients, and negative results were found for 16 patients. The TPHA test was performed on the 16 patients who had negative RPR results, and positive results were found for 15 patients, while a negative result was found for 1 patient.

**Figure 1:**
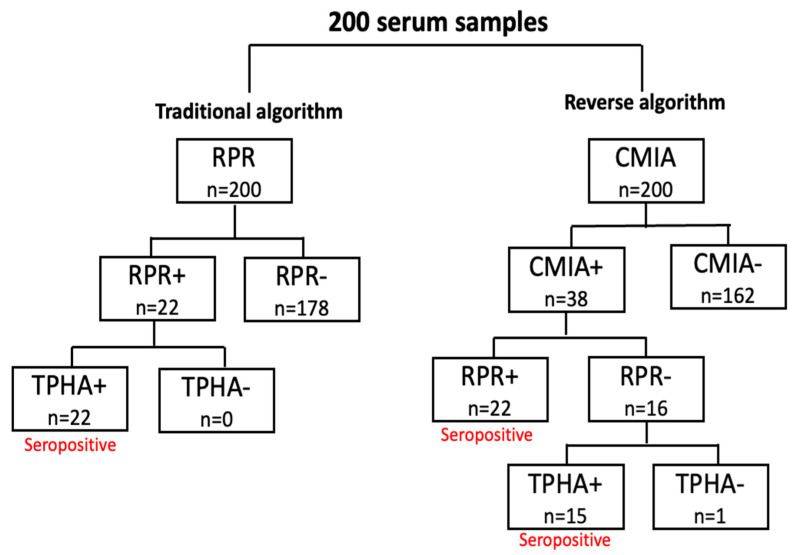
RPR, rapid plasma reagin; TPHA, Treponema Pallidum hemagglutination assay; CMIA, chemiluminescent microparticle enzyme immunoassay.

When these results were evaluated, syphilis coinfection was detected in 22 patients using the traditional algorithm and in 37 patients using the reverse algorithm. In the reverse algorithm group, 15 patients demonstrated a CMIA+RPR−TPHA+ result, likely reflecting past syphilis infections rather than early syphilis cases. These findings suggest that the reverse algorithm detects both active and previously treated or latent syphilis cases, which may complicate the interpretation of results for early syphilis. The patient who gave the CMIA+RPR−TPHA− result was not evaluated as syphilis-positive because a single serological test was insufficient for diagnosis. The results of the tests are shown in Figure 1.

Among the patients who had syphilis coinfection, 25 were evaluated as late latent. Two of these patients had ocular syphilis, and one had neurosyphilis. Ten patients were evaluated as having secondary syphilis, and two patients as having primary syphilis. Among the 10 patients evaluated as having secondary syphilis, 8 had widespread rashes on their body. Among the remaining two patients, one had rashes on the soles of their feet, and the other had rashes on their lower extremities. Lesions were observed on the penis in the two patients in the primary stage.

### 3.3. The Demographic Data of the Patients with HIV/Syphilis Coinfection

Among the 22 patients who were found to be syphilis-positive with the traditional algorithm, 20 were male, 2 were female, 16 were single, and 6 were married. The average age was 34.3. The age group with the most patients was the 26–35 age group, with 10 people. Of the patients, 14 were living in Istanbul and 8 were living in other cities. When sexual orientation was examined, it was found that the homosexual group, with 11 patients, was larger than the heterosexual (7) and bisexual (4) groups. The education levels of the patients were grouped into primary school, secondary school, high school, and university. University was the most common, with 10 patients.

Among the 37 patients who were found to be positive for syphilis with the reverse algorithm, 35 were male, 2 were female, 28 were single, and 9 were married. The average age was 37.2. Parallel to the reverse algorithm, the 26–35 age group was the group with the highest number of patients (*n* = 15). All of the patients resided in Istanbul. When their sexual orientation was examined, the homosexual group was the group with the highest number of patients, as in the traditional algorithm, with 19 patients. Also, 13 were heterosexual, and 5 were bisexual. The education levels of the patients were again grouped into primary school, secondary school, high school, and university. University was the group with the highest number of patients (17 patients), followed by primary school (10), high school (7), and secondary school (3). The demographic data of the patients who had syphilis coinfection are given in Table 2.

### 3.4. The Evaluation of CD4+ and CD8+ T-Lymphocyte Values, CD4/CD8 Rates, and HIV RNA Loads in Patient Groups

Among the 200 people living with HIV who were included in the study, 163 patients without syphilis coinfection were considered to have isolated HIV infection. The mean CD4^+^ T-lymphocyte and mean CD8^+^ T-lymphocyte values of the patients considered to have isolated HIV infection were found to be 534.6 and 997.1 cells/µL, respectively. The mean CD4/CD8 rate was 0.66, and the mean HIV RNA amount was 874.058 copies/mL.

In 22 patients who had HIV/syphilis coinfection detected by using the traditional algorithm, the mean CD4^+^ and mean CD8^+^ T-lymphocyte values were 579.6 and 956.9 cells/µL, respectively. Among the 22 patients, 1 had a CD4^+^ value between 0 and 200 cells/µL, and 9 had a CD4^+^ value above 700 cells/µL. The mean CD4/CD8 rate was 0.75, and the CD4/CD8 rate was below 0.3 in five patients and above 1.0 in five patients. The mean HIV RNA amount was 150,589 copies/mL. Although the HIV RNA amount was below 100 copies/mL in 11 patients, it was above 100,000 copies/mL in 6 patients.

The mean CD4+ and mean CD8+ T lymphocyte values were 538.4 and 996.3 cells/µL, respectively, in 37 patients with HIV/syphilis coinfection detected using the reverse algorithm. The CD4+ T-lymphocyte values of 3 patients were between 0 and 200 cells/µL; the CD4+ T-lymphocyte values of 11 patients were more than 700 cells/µL. The mean CD4/CD8 ratio was 0.68, and the CD4/CD8 rate was below 0.3 in 11 patients and above 1.0 in 7 patients. The mean HIV RNA amount was 346,788 copies/mL. The HIV RNA value was below 100 copies/mL in 13 patients and above 100,000 copies/mL in 16 patients. The CD4+ T-lymphocyte, CD8+ T-lymphocyte, and CD4/CD8 and HIV RNA values of the patients with syphilis coinfection detected by traditional and reverse algorithms are given in Table 3.

## 4. Discussion

Syphilis coinfection has been shown to exacerbate HIV infection by increasing HIV viral load and decreasing CD4+ T-cell counts in HIV-positive individuals. The assessment of serological response to syphilis in people living with HIV (PLWH) presents unique challenges due to the immunological alterations associated with HIV. As highlighted in the study by Marchese et al. (2022) [22], PLWH demonstrate a slower serological response to syphilis treatment compared to HIV-negative individuals. This slower response is particularly significant among patients with an RPR titer > 1:16, which has been identified as an independent predictor of serological non-response. Furthermore, PLWH are more likely to exhibit a serofast status, where non-treponemal test titers remain reactive despite adequate treatment. The serofast condition persists similarly at 6 and 12 months post-treatment, underscoring the need for extended follow-up periods to accurately evaluate treatment outcomes. Serological non-response and serofast status are critical considerations in managing syphilis in PLWH. In our study, we observed that despite effective treatment, some patients exhibited persistently elevated RPR titers, consistent with findings in the literature. Marchese et al. suggest that baseline RPR titers and delayed follow-up assessments may influence the serological outcomes. These findings align with our results, emphasizing the importance of comprehensive serological monitoring over longer durations to detect and address persistent serological abnormalities. Addressing these challenges requires tailored strategies for PLWH. Routine syphilis screening, even in asymptomatic stages, is vital due to the high prevalence of reinfections and the potential for atypical serological responses. Multidisciplinary approaches, integrating updated guidelines and individualized follow-up plans, can optimize diagnostic accuracy and treatment efficacy in this population. Future research should focus on the mechanisms underlying serological non-response and the clinical implications of serofast status to refine management protocols for syphilis in PLWH [22]. Jarzebowski et al. (2006) [23] conducted a study in Paris to assess the impact of syphilis on HIV viral load and CD4+ T-cell count in people living with HIV. This study included 282 male patients diagnosed with syphilis and 1233 HIV-positive male patients without syphilis, with a mean age of 38 years. The results indicated that, within the first six months, 27.3% of patients with syphilis coinfection experienced an increase in HIV viral load, compared to 16.6% in the group without syphilis. The mean increase in viral load was significantly higher in the syphilis-coinfected group (54,000 copies/mL) compared to the non-coinfected group (11,318 copies/mL). Furthermore, a modest decrease in CD4+ T-cell count (approximately −30/μL) was observed during the syphilis episode, which was independently associated with an increase in HIV RNA levels. These findings suggest that immune activation during syphilis infection may facilitate HIV replication, thereby increasing viral load [23].

In contrast, our study found no significant difference in HIV RNA levels or CD4+ T-cell counts between people living with HIV with and without syphilis coinfection. Specifically, the mean HIV RNA viral load in 163 people living with HIV without syphilis was 874,058 copies/mL, with a mean CD4+ T-cell count of 534.6 cells/μL. In contrast, 37 patients with syphilis coinfection had a mean HIV RNA viral load of 346,788 copies/mL and a mean CD4+ T-cell count of 538.4 cells/μL. These results suggest that syphilis coinfection did not significantly affect HIV RNA levels or CD4+ T-cell counts in our cohort.

Buchacz et al. (2006) [24] conducted a study across three clinics in San Francisco and Los Angeles, comparing HIV RNA levels and CD4+ T-cell counts in HIV-positive male patients before and after syphilis infection. This study revealed that syphilis coinfection was associated with an increase in HIV viral load and a decrease in CD4+ T-cell count, likely due to the shared risk behaviors for both infections. The study emphasized the importance of integrated public health efforts to prevent and treat syphilis, which could mitigate the transmission of both HIV and syphilis in the community [24]. However, in our study, we did not observe a significant impact of syphilis coinfection on HIV RNA levels or CD4+ T-cell counts.

Fan et al. (2021) [25] analyzed data from 3829 people living with HIV, including 1539 (40.2%) who were coinfected with syphilis. The study found that the mean CD4+ T-cell count in syphilis-coinfected patients was lower than in those without syphilis (258 vs. 276 cells/μL), with a similar difference observed in the CD4/CD8 ratio. Although the CD4+ T-cell count and the CD4/CD8 ratio are important immunological markers of HIV progression and non-AIDS disease risk, no significant differences were observed in our study in terms of these markers between patients with and without syphilis coinfection (538.4 vs. 534.6 cells/μL and 0.679 vs. 0.659, respectively) [25].

Syphilis screening is typically performed using serological assays to detect both treponemal and non-treponemal antibodies. The order in which these tests are administered distinguishes the traditional algorithm from the reverse algorithm. In the traditional algorithm, a non-treponemal test such as the rapid plasma reagin (RPR) or Venereal Disease Research Laboratory (VDRL) test is used first, followed by confirmation with a treponemal test. The reverse algorithm, however, uses a treponemal test as the initial screening method, followed by a non-treponemal test for confirmation. In cases of discordant results, a second confirmatory treponemal test is performed. It is important to note that no single “gold standard” exists for syphilis serological testing, and results must be interpreted in conjunction with clinical manifestations [26].

Chen et al. (2015) [27] compared the traditional and reverse algorithms for syphilis seropositivity in a cohort of 865 people living with HIV. The reverse algorithm identified 92 additional cases of syphilis compared to the traditional algorithm (24.9% vs. 14.2% positivity) [27]. In our study, similar results were observed: the traditional algorithm detected 11% syphilis seropositivity, while the reverse algorithm identified an additional 7.5% (18.5% positivity overall).

Simčič et al. (2016) evaluated the agreement between the CMIA and TPHA tests in a cohort of 437 serum samples, finding a high level of concordance between the two tests [28]. In our study, we observed similar results, with 37 patients testing positive for both TPHA and CMIA, while 162 patients had negative results for both tests. One patient had a CMIA-positive/TPHA-negative result, further supporting the high agreement between the two tests.

Globally, the epidemiology of syphilis has been significantly influenced by the HIV epidemic. The relationship between these two diseases became apparent in the early 1980s, with syphilis rates rising among men who have sex with men (MSM) during the HIV epidemic. Recent data from Turkey (2018) indicated a higher prevalence of syphilis among MSM (28.7%) compared to heterosexual individuals (14.7%) [29,30]. In our study, the majority of syphilis-positive patients, as detected by both the traditional and reverse algorithms, were MSM, which aligns with the literature.

In a previous study by Köksal et al. (2016) [30], syphilis seroprevalence in HIV-positive men was found to be 19.3%, with MSM showing a significantly higher seroprevalence (28.7%) compared to heterosexual men [30]. Our study similarly found a syphilis seroprevalence of 18.5%, with MSM representing the largest affected group.

There are studies in the literature evaluating traditional and reverse algorithms in the diagnosis of syphilis infection. However, studies evaluating the performance of these algorithms in the case of HIV and syphilis coinfection are limited. Therefore, the present study aimed to contribute to filling this important gap in the literature. Although there was a decrease in the number of syphilis cases and incidence rate in Turkey in 2020 due to the impact of the COVID-19 pandemic, an increase in the number of cases and incidence rate was observed between 2021 and 2023. These data from the Ministry of Health of the Republic of Turkey reveal that syphilis infection poses a significant threat to public health in Turkey [11]. Our study showed that the reverse algorithm detected more positivity in the diagnosis of syphilis. Considering this increase in the number of cases and incidence rate in Turkey, it is clear that the reverse algoristhm will contribute to the earlier diagnosis of syphilis coinfection in HIV-positive individuals. This provides a significant benefit in terms of both improving individual treatment processes and protecting public health.

The reverse algorithm detected 7.5% more positive results compared to the traditional algorithm. However, this increase includes cases with CMIA+RPR−TPHA+ profiles, which are likely indicative of past infections rather than active early syphilis. Therefore, while the reverse algorithm shows higher sensitivity in identifying syphilis exposure, its broader detection spectrum also highlights potential limitations in distinguishing active infections from previously treated cases. This nuance should be considered when interpreting the algorithm’s diagnostic performance and its applicability in different clinical scenarios. Future studies could focus on refining the diagnostic criteria to better differentiate between active and past syphilis infections.

Our study has several limitations. First, data such as the reinfection rate and serofast status of the patients could not be obtained. Second, information on whether patients were using other antimicrobial medications at the time of follow-up visits was not available. Additionally, since it is a single-center study, it does not reflect all the data available in Turkey. Nevertheless, it is important in terms of providing important epidemiologic data for these patients.

## 5. Conclusions

Our findings suggest that syphilis coinfection does not significantly impact HIV viral loads, CD4+ T-cell counts, or CD4/CD8 ratios in people living with HIV. These results highlight the need for continued surveillance and integrated public health strategies to address both syphilis and HIV, particularly in high-risk populations such as MSM.

In the present study, the reverse algorithm detected 7.5% more syphilis seropositivity compared to the traditional algorithm. This finding underscores the enhanced diagnostic utility of the reverse algorithm for syphilis detection. Given its superior performance, we advocate for the use of the reverse algorithm in people living with HIV, where syphilis coinfection is of critical concern due to its potential impact on disease progression. Our results are consistent with those of several previous studies conducted globally, further supporting the efficacy of the reverse algorithm in identifying syphilis coinfection in people living with HIV.

However, it is important to note that each laboratory should select the algorithm that best aligns with its resources, capacity, and specific needs. Considering the shared transmission routes of syphilis and HIV, we recommend routine screening and monitoring for syphilis, as well as other sexually transmitted infections, in people living with HIV at regular intervals. This approach would facilitate early detection and intervention, ultimately improving patient outcomes.

## Figures and Tables

**Table 1:** The demographic data of the people living with HIV included in the study.

Demographic Data		*n* (%)
Sex	Male	168 (84)
	Female	32 (16)
Residence	Istanbul	170 (85)
	Other cities	30 (15)
Age groups	18–25	29 (14.5)
	26–35	55 (27.5)
	36–45	54 (27.0)
	46–55	38 (19.0)
	>55	24 (12.0)
Sexual orientation	Heterosexual	124 (62)
	Homosexual	60 (30)
	Bisexual	15 (7.5)
	Transgender	1 (0.5)
Education level	Primary education	64 (32)
	Secondary education	8 (4)
	High school	58 (29)
	University	70 (35)
Marital status	Married	89 (44.5)
	Single	111 (55.5)

**Table 2:** The demographic data of the patients with HIV/syphilis coinfection.

		Traditional Algorithm			Reverse Algorithm		
		Positive	Negative		Positive	Negative	
		*n* (%)	*n* (%)	*p* Value	*n* (%)	*n* (%)	*p* Value
Sex	Male	20 (90.9)	148 (83.1)	0.318	35 (94.6)	133 (81.6)	0.052
	Female	2 (9.1)	30 (16.9)		2 (5.4)	30 (18.4)	
Age group	18–25	4 (18.2)	25 (14)	0.208	5 (13.5)	24 (14.7)	0.208
	26–35	10 (45.5)	45 (25.3)		15 (40.5)	40 (24.5)	
	36–45	5 (22.7)	49 (27.5)		9 (24.3)	45 (27.6)	
	46–55	2 (9.1)	36 (20.2)		3 (8.1)	35 (21.5)	
	≥56	1 (4.5)	23 (12.9)		5 (13.5)	19 (11.7)	
Residence	Istanbul	14 (63.6)	156 (87.6)	0.008 *	27 (73)	143 (87.7)	0.023 *
	Other cities	8 (36.4)	22 (12.4)		10 (27)	20 (12.3)	
Sexual orientation	Heterosexual	7 (31.8)	117 (65.7)	0.017 *	13 (35.1)	111 (68.1)	0.003 *
	Homosexual	11 (50)	49 (27.5)		19 (51.4)	41 (25.2)	
	Bisexual	4 (18.2)	11 (6.2)		5 (13.5)	10 (6.1)	
	Transgender	0 (0)	1 (0.6)		0 (0)	1 (0.6)	
Education level	Primary education	3 (13.6)	61 (34.3)	0.027 *	10 (27)	54 (33.1)	0.144
	Secondary education	3 (13.6)	5 (2.8)		3 (8.1)	5 (3.1)	
	High school	6 (27.3)	52 (29.2)		7 (18.9)	51 (31.3)	
	University	10 (45.5)	60 (33.7)		17 (45.9)	53 (32.5)	
Marital status	Married	6 (27.3)	83 (46.6)	0.085	9 (24.3)	80 (49.1)	0.006 *
	Single	16 (72.7)	95 (53.4)		28 (75.7)	83 (50.9)	

*: Significant at *p* < 0.05 level according to Chi-squared and Bonferroni tests.

**Table 3:** Laboratory parameters of the patient groups.

		Traditional Algorithm			Reverse Algorithm		
		Positive	Negative		Positive	Negative	
		*n* (%)	*n* (%)	*p* Value *	*n* (%)	*n* (%)	*p* Value *
CD4 cells/(µL)	0–200	1 (4.5)	41 (23)	0.136	3 (8.1)	39 (23.9)	0.148
	201–350	3 (13.6)	19 (10.7)		6 (16.2)	16 (9.8)	
	351–500	6 (27.3)	30 (16.9)		10 (27)	26 (16)	
	501–700	3 (13.6)	36 (20.2)		7 (18.9)	32 (19.6)	
	>700	9 (40.9)	52 (29.2)		11 (29.7)	50 (30.7)	
CD8 cells/(µL)	0–350	0 (0)	16 (9)	0.298	0 (0)	16 (9.8)	0.115
	351–600	6 (27.3)	33 (18.5)		9 (24.3)	30 (18.4)	
	601–900	7 (31.8)	52 (29.2)		11 (29.7)	48 (29.4)	
	901–1500	6 (27.3)	59 (33.1)		12 (32.4)	53 (32.5)	
	>1500	3 (13.6)	18 (10.1)		5 (13.5)	16 (9.8)	
CD4\CD8	<0.3	5 (22.7)	56 (31.5)	0.777	11 (29.7)	50 (30.7)	0.576
	0.3–0.5	3 (13.6)	30 (16.9)		5 (13.5)	28 (17.2)	
	0.51–0.7	4 (18.2)	27 (15.2)		9 (24.3)	22 (13.5)	
	0.71–1.0	5 (22.7)	24 (13.5)		5 (13.5)	24 (14.7)	
	>1.0	5 (22.7)	41 (23)		7 (18.9)	39 (23.9)	
HIV RNA (copies/mL)	≤100	11 (50)	76 (42.7)	0.590	13 (35.1)	74 (45.4)	0.477
	101–1000	1 (4.5)	2 (1.1)		1 (2.7)	2 (1.2)	
	1001–10,000	0 (0)	4 (2.2)		0 (0)	4 (2.5)	
	10,001–100,000	4 (18.2)	30 (16.9)		7 (18.9)	27 (16.6)	
	>100,000	6 (27.3)	66 (37.1)		16 (43.2)	56 (34.4)	

*: Significant at *p* < 0.05 level according to Chi-squared and Bonferroni tests.

## Data Availability

Datasets are available on request from the authors.
